# Antimicrobial Polymeric Surfaces Using Embedded Silver Nanoparticles

**DOI:** 10.3390/antibiotics12020207

**Published:** 2023-01-18

**Authors:** Pooja Sharma, Luisa Fialho, Nuno Miguel Figueiredo, Ricardo Serra, Albano Cavaleiro, Sandra Carvalho

**Affiliations:** 1 CEMMPRE, Mechanical Engineering Department, University of Coimbra, 3030-788 Coimbra, Portugal; 2 CFUM-UP, Centro de Física das Universidades do Minho e do Porto, University of Minho, Campus of Azurém, 4800-058 Guimaraes, Portugal; 3 i3S—Instituto de Investigação e Inovação em Saúde, Universidade do Porto, Rua Alfredo Allen, 208, 4200-135 Porto, Portugal; 4 INEB—Instituto de Engenharia Biomédica, Universidade do Porto, Rua Alfredo Allen, 208, 4200-135 Porto, Portugal; 5 IPN—LED&MAT, Instituto Pedro Nunes, Rua Pedro Nunes, 3030-199 Coimbra, Portugal

**Keywords:** polycarbonate, silver nanoparticles, thermal embedding, glass transition temperature, antimicrobial activity

## Abstract

Pathogens (disease-causing microorganisms) can survive up to a few days on surfaces and can propagate through surfaces in high percentages, and thus, these surfaces turn into a primary source of pathogen transmission. To prevent and mitigate pathogen transmission, antimicrobial surfaces seem to be a promising option that can be prepared by using resilient, mass-produced polymers with partly embedded antimicrobial nanoparticles (NPs) with controlled size. In the present study, a 6 nm thick Ag nanolayer was sputter deposited on polycarbonate (PC) substrate and then thermally annealed, in a first step at 120 °C (temperature below Tg) for two hours, for promoting NP diffusion and growth, and in a second step at 180 °C (temperature above Tg) for 22 h, for promoting thermal embedding of the NPs into the polymer surface. The variation in the height of NPs on the polymer surface with thermal annealing confirms the embedding of NPs. It was shown that the incorporation of silver nanoparticles (Ag NPs) had a great impact on the antibacterial capacity, as the Ag NP-embedded polymer surface presented an inhibition effect on the growth of Gram-positive and Gram-negative bacteria. The tested surface-engineering process of incorporating antimicrobial Ag NPs in a polymer surface is both cost-effective and highly scalable.

## 1. Introduction

Infectious diseases (IDs) are a leading cause of death worldwide and affect so many people globally per year. Pathogens (bacteria, fungi and viruses), including virulent and drug-resistant strains, have consistently been found in high percentages on surfaces all over the world [1,2]. Most pathogens are able to survive on surfaces for up to several months that can act as primary sources of pathogen transmission if no disinfection is performed [3,4,5]. This transmission of pathogens through inanimate surfaces and equipment is a crucial challenging health problem in intensive care units all over the world [5]. Another common example is the transmission of impetigo (a very contagious skin disease that affects mostly children) through contaminated toys [6].

To prevent this surface transmission of diseases, novel multifunctional antiviral/antimicrobial surfaces need to be developed. Silver nanoparticles (Ag NPs) have been shown to exhibit broad-spectrum antiviral/antimicrobial properties, including against virulent and drug-resistant strains [7]. Although they have been associated with health hazards [7], when released in small concentrations, Ag is non-toxic to humans, leading to an increased interest in their application in medical devices [8,9], and also being highlighted as an anti-inflammatory agent [7]. Ag NPs have also been investigated as a potential anticancer agent [10,11]. In vivo assays demonstrate the effectiveness of Ag NPs on antimicrobial activity, epithelization, and collagen deposition [12]. Ag antimicrobial activity mechanism may occur in various forms such as ionic, reactive oxygen species (ROS), or by NPs internalization, depending on the embedding matrix [7,13,14].

Further extension of applications of biocide metals can be made possible via polymer/metal composites. A large percentage of the steady growth of the polymer market is due to their interesting properties, such as stiffness/flexibility, opacity/transparency, durability, barrier behavior for gases and liquids, chemical and heat resistance, and low production cost [15]. This set of properties has unlocked their extensive use in several industries and applications, including in medical devices, packaging products, and delivery systems for solid and liquid pharmaceuticals [15]. Thus, the development and deployment of antimicrobial polymers could be a highly desirable strategy to avoid surface transmission of pathogens. Such polymers could be prepared either by embedding a biocide agent into the bulk polymer or by stabilizing bioactive NPs over the polymeric surfaces [16,17].

Polycarbonate (PC) is a transparent thermoplastic with carbonate functional groups, which can be melted and forced into a mold with high pressure to give it the desired shape. It has remarkable mechanical and optical properties, being resistant to impact and fracture [18]. Moreover, it may show eco-friendly processing and recyclability. Due to its desirable characteristics, PC is extensively used worldwide in products such as water bottles, monitor screens, aircraft interiors, screens, cover sheets, automotive light covers, etc. [18,19,20]. Therefore, the development of a post-processing, affordable, scalable method for making PC surfaces antimicrobial might have a great positive impact on human health and safety.

The direct deposition of NPs with biocide activity over the surface of materials is an efficient way to provide it with antiviral/antimicrobial functionality. Physical, chemical, and biological methods can be used to synthesize such NPs [10,11,13]. However, the deposited NPs usually show very low adhesion and, hence, can easily agglomerate and/or be lost to the environment by simple mechanical action. A promising method to stabilize NPs on a surface consists of the thermal embedding of NPs over an amorphous substrate [21]. Per surface science, the immersion on a rigid metal NP of a soft material can occur when the surface tension of the NP/air interface is greater than the sum of the surface tensions at the matrix/air and NP/matrix interfaces [22,23,24]. Considering our system, the surface tension of Ag (1200 mJ/m^2^) is significantly higher than that of PC (30–40 mJ/m^2^) [25,26]. However, in the case of a solid-state polymer such as PC, the NP embedding requires a change in Gibbs free energy to promote the work of adhesion. This energy can be provided by external heating (thermal annealing) at a temperature near the thermal softening temperature (Tg) of the amorphous material, leading to an increase in polymer chain mobility at the polymer/air interface and facilitating the indentation [20,27].

In this study, PC polymeric surfaces were coated with Ag NPs and subsequently thermally annealed below and above the substrate’s Tg. The samples were characterized concerning surface morphology, NP adhesion, reflectivity and antibacterial properties.

## 2. Materials and Methods

### 2.1. Ag Nanoparticles Deposition

An Ag nanolayer with a nominal thickness of ~6 nm was deposited using a pure (99.99%) Ag planar target (50.8 mm in diameter by 3 mm in thickness) in a pure (99.999%) Ar atmosphere. Ar flux in the inlet was set to 30 sccm, corresponding to a constant deposition pressure of 0.3 Pa. The target-to-substrate distance was maintained at 12 cm, and the rotation speed of the substrate holder was set to 30 rpm. The substrate holder was kept at a floating potential. PC substrates of 10 × 10 × 5 mm were used. Preceding the depositions, the PC substrates were cleaned in an ultrasonic bath, in two steps using different solvents: ethanol, and distilled water, each one for 10 min. A DC power supply (Advanced Energy Pinnacle Plus, CO, USA) was used with a constant power of 100 W during deposition. The deposition chamber was pumped with rotary (Pfeiffer Vacuum, Germany DUO 20 M, pumping speed 20 m^3^/h) and diffusion (BOC Edwards Diffstak, England 160/700, pumping speed 2736 m^3^/h) pumps to a base pressure below 5 × 10^−4^ Pa.

### 2.2. Thermal Annealing Treatments

In thermogravimetric (TG) measurements, a Netzsch TG 209 F1 Libra was used, where a nitrogen flow of 20 mL/min was used, with a heating ramp at 10 K/min from 30 to 600 °C. Regarding the conditions defined in the differential scanning calorimetry (DSC) measurements, a nitrogen flow of 40 mL/min and a heating rate of 20 K/min between −95 and 300 °C was implemented. The equipment used was the Netzsch DSC 204 F1 Phoenix. For data processing of results, the Proteus 8.0^®^ program was used. DSC was performed on the PC substrate to determine its glass transition temperature (Tg). Based on this observation, thermal annealing of the deposited substrates was performed in a two-stage sequence: (i) in the first step, the substrates were annealed in the oven for two hours at 120 °C (a temperature below the substrate Tg) to promote thermal dewetting of the Ag nanolayer and NP formation; (ii) in a second step, these samples were subsequently heated for 22 h at 180 °C (a temperature above the substrate Tg) to promote NP growth and NP thermal embedding.

### 2.3. General Characterizations

In this work, in order to test the adhesion of the coatings/NPs to the polymeric substrates, 1 cm viton balls were adapted into a scratch tester stylus to perform scratch tests using a progressive load setup up to 15 N. The vertical load rate was set to 100 N/min, whereas the horizontal velocity was set to 30 mm/min. Experiments were performed in a CSEM Revetest automatic scratch tester.

The surface morphology of samples was analyzed by atomic force microscopy (AFM) on a Bruker diInnova using a silicon tip with 6 nm of tip radius. The scan area for all films was 5 × 5 µm^2^. Grain size, shape, and height distributions were obtained for each sample using the open-source software Gwyddion 2.61. For the reflectivity measurement, the device used was a portable spectrophotometer of the brand ColorEye^®^ XTH, model Greta Macbeth with SCI (specular component included). The reflectivity values are given as a function of the wavelength of the light, corresponding in this case to a range between 360 and 750 nm, with an increment of 10 nm between the points.

### 2.4. Antibacterial Assessment

The antibacterial activity of the films was tested using two bacteria, a Gram-positive *Staphylococcus aureus* (ATCC 6538) and a Gram-negative *Escherichia coli* (CECT 434). The zone of inhibition (ZoI) or halo test, adapted from the Kirby–Bauer method, was performed to evaluate the antibacterial activity. Firstly, the samples were esterized by exposure to UV light for 1 h. The inoculum of each bacterium was prepared by inoculation of a single colony in 30 mL of Tryptic Soy Broth (TSB, Frilabo, Milheirós, Portugal) and incubated at 37 °C and 120 rpm overnight. The optical density (OD) of the inoculum was measured at 620 nm and properly diluted in culture media to 1 × 108 CFU/mL. An aliquot of cellular suspension (100 μL) was spread in Tryptic Soy Agar (TSA, Merck, Darmstadt, Germany) Petri dishes. After medium solidification, the samples were placed separately on the top of the agar plate, placed on the side with the film in contact with the agar, and incubated for 24 h at 37 °C. After the incubation period, the halo (transparent medium zone, which translates that there is no bacteria growth) formed around the samples was photographed to record the results (images captured by Image LabTM software 3.0). All the experiments were repeated with at least three independent assays. After the halo test, the samples were removed and washed three times in distilled water, followed by sequential dehydration in graded ethanol solutions (70, 95, and 100% (*v*/*v*) for 10, 10, and 20 min, respectively), and stored in a desiccator. Then, the cultured samples were coated with an Au/Pd thin film by sputtering using the SPI Module Sputter Coater equipment and analyzed by SEM (Quanta 400 FEG ESEM/EDAX Genesis X4M, Thermo Fisher Scientific, Waltham, MA, USA).

## 3. Results and Discussion

### 3.1. DSC and TG

**Figure 1:**
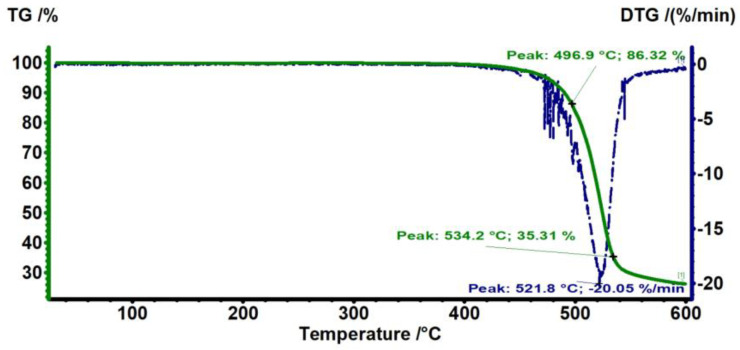
TG thermograms of PC, where TG % is the mass percentage of the polymer sample remaining after heating the polymer to a certain temperature.

Figure 1 shows the TG/DTG analysis of the PC substrate. No change in mass is observed at around 100–200 °C, indicating that the PC is free from any absorbed solvent or moisture. The major mass loss of around 80% occurred from 400 to 550 °C, which is related to the decomposition of the polymer matrix. The PC starts to degrade from approximately 440 °C and degraded up to 86.3% in mass at 496.9 °C. As the temperature increases further, the mass loss increases up to ~35.3% at 534.2 °C. These temperatures are close to those reported in the literature for the degradation temperature of PC [28].

**Figure 2:**
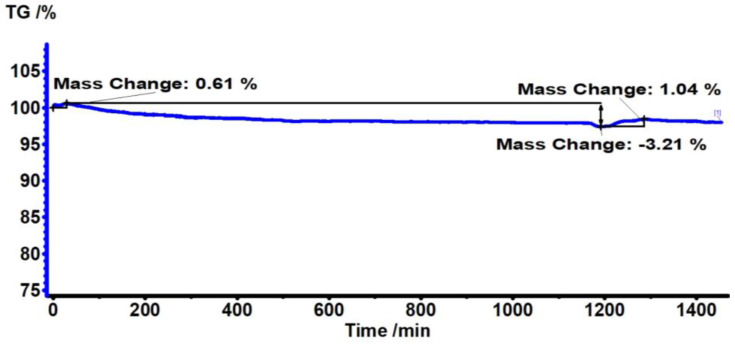
TG thermograms of PC substrate heated at 180 °C for 24 h, where TG % is the mass percentage of the polymer sample remaining after heating the polymer to a certain temperature.

Figure 2 shows the TG/DTG analysis of the PC substrate, made during 24 h at 180 °C. The results indicate that there is no significant mass loss within this time period. It can be seen that there is only about a 3% percent mass loss, which states the stability of the PC polymer during thermal annealing at 180 °C for 24 h.

The mechanical and physical properties of an amorphous or semi-crystalline polymer are dependent on the behavior of the temperature, which is defined by the glass transition temperature. When this Tg is reached, the transition of the amorphous region from a rigid state to a more flexible and ductile state takes place. When the temperature is below Tg, the molecular chains of amorphous materials are not mobile. On the other hand, at a temperature above the Tg, the amount of energy supplied to the material allows these chains to acquire mobility, exhibiting a “rubbery” behavior [29].

**Figure 3:**
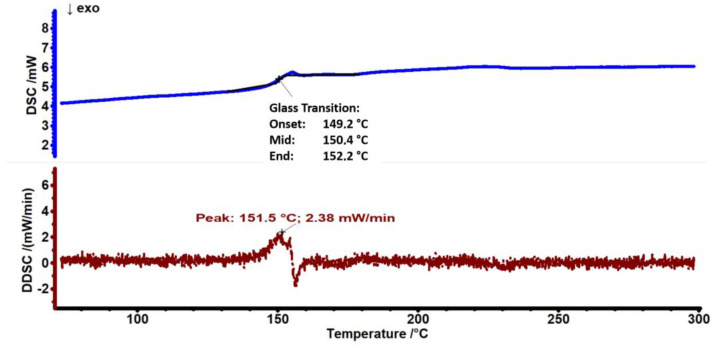
DSC and DDSC thermograms of PC substrate.

DSC characterization was performed to determine the glass transition temperature (Tg) of PC polymers, as shown in Figure 3. The DSC curve of the PC in the heating cycle exhibits a glass transition temperature of ≈150.4 °C. No amorphous polymer can exhibit a melting transition, as melting is a first-order transition occurring only for crystalline polymers [27].

### 3.2. Surface Morphology

**Figure 4:**
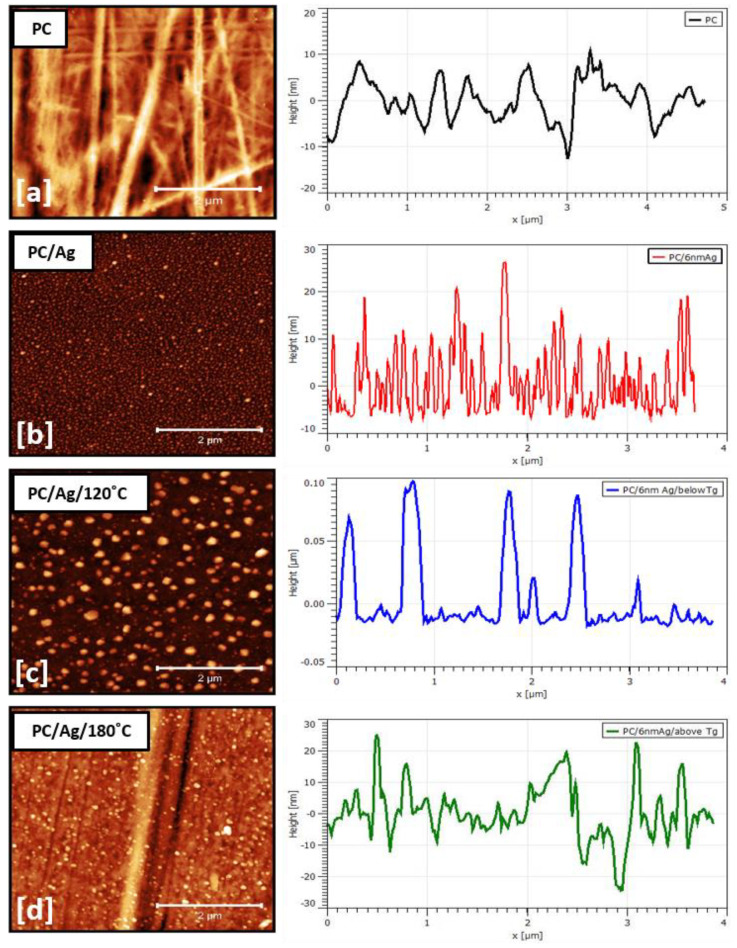
AFM surface micrograph and line profile of sample: (**a**) PC; (**b**) PC/Ag; (**c**) PC/Ag/120 °C; (**d**) PC/Ag/180 °C.

**Figure 5:**
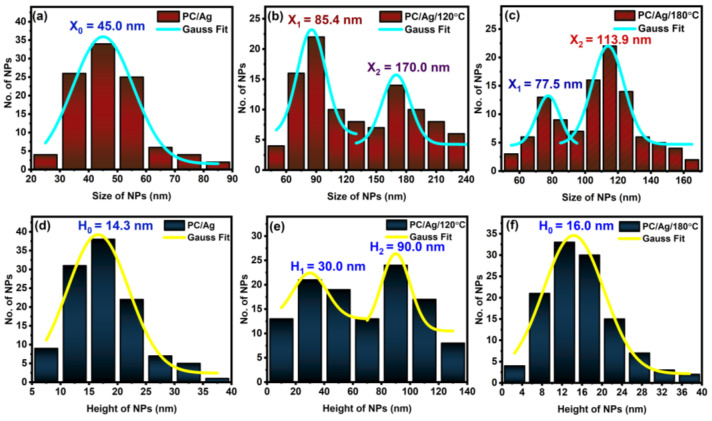
Ag NPs’ size distribution curves for sample: (**a**) PC/Ag; (**b**) PC/Ag/120 °C; (**c**) PC/Ag/180 °C and Ag NPs’ height distribution curves for sample (**d**) PC/Ag; (**e**) PC/Ag/120 °C; (**f**) PC/Ag/180 °C.

The surface morphology of samples was examined by AFM, as shown in Figure 4. Calculated surface analysis parameters, such as NP lateral size and height distribution curves, are shown in Figure 5. Figure 4a,b are shown the two-dimensional AFM images of the pure and Ag-coated PC substrates, respectively. Despite the nominal thickness of the Ag coating being around 6 nm, such an amount was clearly below the percolation limit for this system, originating in the presence of discontinuous, well-separated small islands on the surface of the substrate. For the as-deposited sample, the average NP diameter was 45 nm, and the average NP height was 14 nm (Figure 5a). After the thermal annealing treatment at 120 °C, the nanoparticles increased in both lateral size and height, developing bimodal size/height distribution curves centered at around 85/30 nm and 170/90 nm (Figure 5b), respectively. This can be explained by the thermally activated surface diffusion and coalescence of bigger nanoparticles along with the nucleation and growth of newer nanoparticles from smaller islands and/or individual atoms. Interestingly, after an additional thermal annealing step at 180 °C (temperature above the substrate Tg, determined by DSC to be 150 °C) both bimodal size/height distribution curves were centered at lower values, close to 78/16 nm and 114/16 nm (Figure 5c). These results, in particular the decreasing NP heights, demonstrate the successful thermal embedding of Ag nanoparticles into the PC surface. If there was no surface embedding process taking place, the combined 120 °C + 180 °C thermal annealing process would obviously generate bigger nanoparticles than the single 120 °C thermal annealing process (due to more energy being given to the system), which is not the case. On a different perspective, when annealing above the substrate Tg, both the NP growth and surface embedding process can take place over time, although most of the metal NP diffusion and growth occurs during the first 15–30 min of annealing, after which the surface embedding process becomes dominant [30]. Thus, even considering that the thermal embedding process was dominant during the 24 h annealing treatment above Tg, some extra energy was still given to the nanoparticles that could translate into some NP growth. We can justify the fact that bigger NP sizes were observed in sample PC/Ag/120 °C than in sample PC/Ag/180 °C by the diffusion and growth of NPs that naturally takes place, even at room temperature, for the case of un-stabilized Ag nanostructures over surfaces [31] (i.e., between the thermal annealing treatment and the SEM analysis, diffusion and growth occurred for the 120 °C condition but not for the 120 + 180 °C condition where the NPs were stabilized). Thus, the surface-embedded Ag NPs have the advantage of being stable over time, in different environments, and even when subject to mechanical contact action (as will be demonstrated ahead in Section 3.4).

### 3.3. Reflectivity

The optical properties of a nanocomposite containing metal NPs are an important, non-destructive way of accessing the information on the NPs’ morphology and environment.

**Figure 6:**
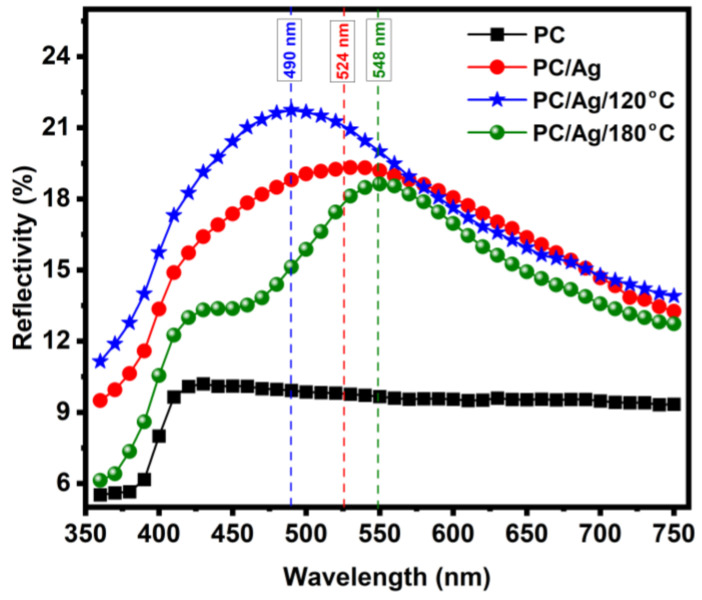
Reflectivity spectra of the pure and coated substrates, before and after each annealing step.

Figure 6 shows the reflectivity plot of pristine and coated PC substrates. All the coated samples showed the localized surface plasmon resonance (LSPR) scattering bands typical of Ag nanoparticles of sizes above 30–40 nm [32]. In PC/6 nm Ag sample (as-deposited film), the spectrum shows a broad LSPR band centered around 524 nm. Such broad red-shifted LSPR bands are characteristic of an ensemble of ellipsoidal nanoparticles having low aspect ratios and/or wide distribution of sizes/shapes [33]. From the optical results alone, we can conclude that the nanolayer of 6 nm is mostly discontinuous, as was attested before by AFM analysis. After annealing at 120 °C for 2 h, a narrowing and a blue-shift of the LSPR scattering band to ~490 nm due to NP shape renormalization (a tendency of the particles to become more spherical) is observed [34], confirming previous AFM observations on the general increase in NPs’ aspect ratio. Further annealing above Tg causes a pronounced narrowing and red-shift of the LSPR scattering band to 548 nm. The narrowing of the LSPR peak can be understood as a narrowing of the NP size and shape distribution curves [34,35] caused by NP diffusion and recrystallization. On the other hand, the pronounced red-shift of the LSPR peak is a confirmation of the NP thermal embedding process. As the NPs penetrate the surface of the polymer, their average surrounding refractive index increases, which causes the buildup of polarization charges that weakens the total restoring force on the dielectric side of the interface, resulting in a lower energy (red-shifted) LSPR scattering peak [35]. Therefore, these optical results are well consistent with the idea of thermally embedded NPs in the PC/Ag/180 °C case.

### 3.4. Adhesion Test

**Figure 7:**
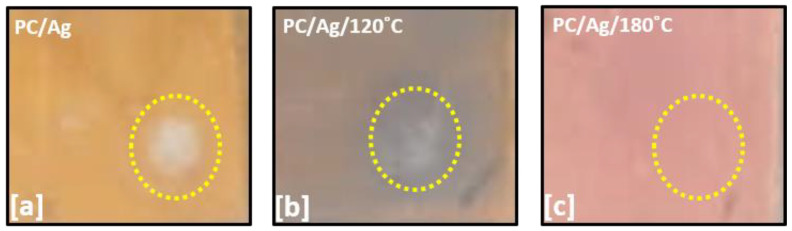
Pictures of the scratch-tested areas of the sample: (**a**) PC/Ag; (**b**)PC/Ag/120 °C; (**c**) PC/Ag/180 °C. Yellow dotted circles were added around the tested zones to facilitate interpretation.

The adhesion of coatings to substrates is an important physical characteristic for evaluating the performance and reliability of coated components. Several techniques allow to check the adhesion of the coating-substrate: the tape test, indentation test, scratch test, laser fragmentation, etc. [36]. In order to test the adhesion of NPs/nanolayer to the PC surface, 1 cm viton balls were adapted into a scratch tester stylus in order to perform scratch tests using a progressive load setup up to 15 N. Such setup was created to mimic the contact-slide of a human finger over a surface. Visual pictures of the tested zones are shown in Figure 7 for the as-deposited, 120 °C-annealed and 180 °C-annealed PC/Ag samples.

As expected, the as-deposited sample was easily scratched due to the low adhesion of the 6 nm Ag nanolayer/nanoparticles to the substrate (white zone circumscribed by a yellow circle in Figure 7a). The 120 °C-annealed sample, containing Ag in the form of bigger NPs, showed better adhesion results but still showed some degree of NP delamination from the polymer surface (Figure 7b). For the 180 °C annealed sample (PC/Ag/180 °C), containing thermally embedded Ag nanoparticles, no delamination zone was observed (Figure 7c), demonstrating the highly robust nature and stability of the multifunctional nanocomposite surfaces obtained by thermal embedding of Ag NPs into PC.

### 3.5. Antibacterial Activity

**Figure 8:**
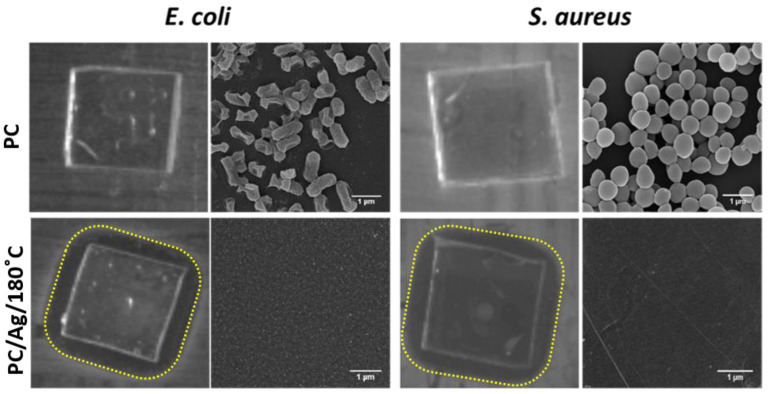
Antibacterial activity against *E. coli* and *S. aureus* of uncoated polymeric surface and polymeric surfaces coated Ag NPs followed by thermal annealing treatment at 180 °C (PC/Ag/180 °C), evaluated by the halo test and SEM micrographs of samples after the halo test (scale bar: 1 μm).

Taking into consideration the best result from the adhesion test (thermally annealed sample), the antibacterial activity of sample PC/Ag/180 °C was evaluated, using the uncoated polymeric surface as control, by the zone of inhibition assays (halo test) and SEM analysis after the halo test against *E. coli* and *S. aureus* (Figure 8).

As expected, the uncoated polymeric surface did not show any effect on both *E. coli* and *S. aureus* bacteria. The sample with thermally embedded Ag NPs (PC/Ag/180 °C sample) presented an inhibition effect on the growth of both bacteria, as an inhibition halo (transparent biological medium with no bacteria growth) was observed around the samples. The SEM micrographs of the samples demonstrated a vast bacteria colonization on the uncoated polymeric surface (PC samples) and a total absence of bacteria on the polymeric surface with thermally embedded Ag NPs (PC/Ag/180 °C sample).

## 4. Conclusions

Robust, mechanically stable nanocomposite PC/Ag surfaces were successfully obtained by sputter depositing an Ag nanolayer over PC, followed by thermal annealing at a temperature above the glass transition point of the substrate. With AFM analysis, it was evident that there was a growth of NPs taking place after annealing at 120 °C, confirming the thermally activated diffusion and growth process. Similarly, the height variation of NPs with thermal annealing at 180 °C (above Tg) confirmed the embedding of the NPs on the PC surface. Reflectivity results were consistent with the idea of NP embedding and shape renormalization at this temperature as well. The adhesion test demonstrated that the thermal embedding process resulted in better adhesion of NPs to the surface.

The Ag NP-embedded polymer surfaces (PC/Ag/180 °C) showed strong antimicrobial activity on *E. coli* and *S. aureus* bacteria, making them antimicrobial surfaces.

Overall, we have demonstrated a cost-effective approach for creating robust antimicrobial surfaces on mass-produced polymers.

## Data Availability

Not applicable.
